# Sclerotherapy Induced Takotsubo Syndrome

**DOI:** 10.1155/2020/5626078

**Published:** 2020-01-14

**Authors:** Sunny Patel, Sepideh Nabatian, Michael Goyfman

**Affiliations:** ^1^Department of Medicine, Long Island Jewish Forest Hills, Northwell Health, New York, USA; ^2^Department of Cardiology, Long Island Jewish Forest Hills, Northwell Health, New York, USA

## Abstract

A 66-year-old female was brought to the emergency department for acute-onset left-sided chest pain. Prior to arrival, she was at an outpatient appointment with a vascular surgeon for elective sclerotherapy treatment of her lower extremity varicose veins. After receiving an IV injection of polidocanol, she developed severe chest pain with left arm and jaw numbness for the first time in her life. Upon arrival to the ED, the patient reported that her symptoms had resolved. Electrocardiogram (ECG) on presentation was significant for T-wave inversions in leads V1-V3. An initial set of cardiac enzymes showed a troponin I level of 0.62 ng/mL, which subsequently increased to 2.26 ng/mL. Her echocardiogram was significant for mild left ventricular systolic dysfunction with apical hypokinesis (ejection fraction 50%). A repeat ECG showed new T-wave inversions compared to that from the time of admission. The patient eventually agreed to cardiac catheterization, which revealed patent vessels without coronary artery disease, supporting our diagnosis of Takotsubo syndrome and what is the first reported case of likely polidocanol-induced Takotsubo syndrome in the United States.

## 1. Introduction

Takotsubo syndrome is typically caused by significant emotional or physical stress and is considered nonischemic in nature. However, the condition can be diagnosed in patients with less typical presentations and histories as well. We present a case of a 66-year-old female who presented to the ED with severe chest pain and was later diagnosed with Takotsubo syndrome after being administered polidocanol, a commonly used sclerotherapy agent.

## 2. Case Report

A 66-year-old female with no significant past medical history was brought to the emergency department by emergency medical services (EMS) with a complaint of left-sided chest pain. She was in her usual state of health when she went to a scheduled appointment with a vascular surgeon for outpatient elective sclerotherapy for the cosmetic treatment of varicose veins. Shortly after being administered an injection of polidocanol, she began experiencing severe left-sided chest pain radiating to her left back with left arm numbness. She also complained of nausea, one episode of vomiting, pressure in her jaw, and lightheadedness. Prior to being transported to the emergency department by ambulance, she was given two doses of aspirin 81 mg.

**Figure 1:**
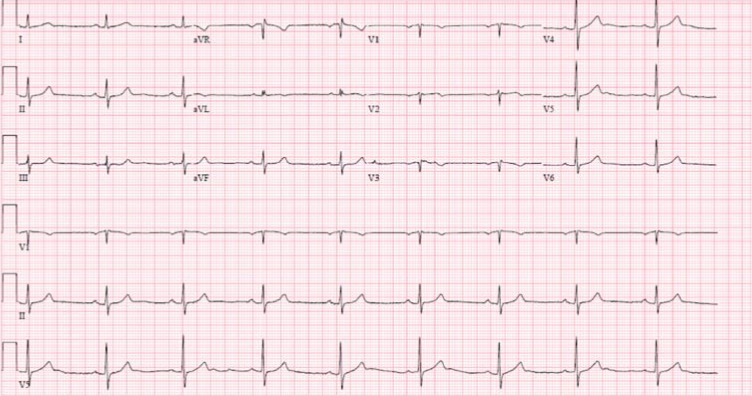
Initial ECG showing T-wave inversions in leads V1-V3.

On arrival to the emergency department, she reported that her symptoms had subsided. Her initial electrocardiogram (ECG) was remarkable for T-wave inversions in leads V1-V3 and sinus bradycardia at 54 beats per minute (Figure 1), with no prior ECG available for comparison. No ST-segment abnormalities were present. Initial laboratory studies were also notable for an elevated troponin I level of 0.62 ng/mL.

The patient was admitted to the inpatient telemetry unit for suspected acute coronary syndrome. Repeat assessment of her cardiac enzymes six hours after her initial bloodwork revealed that her troponin I level had risen to 2.26 ng/mL, at which point the patient had been started on continuous unfractionated intravenous heparin. She remained asymptomatic. The patient was offered cardiac catheterization but initially declined. In a third set of cardiac enzymes, her troponin I level had trended down to 1.15 ng/mL.

**Figure 2:**
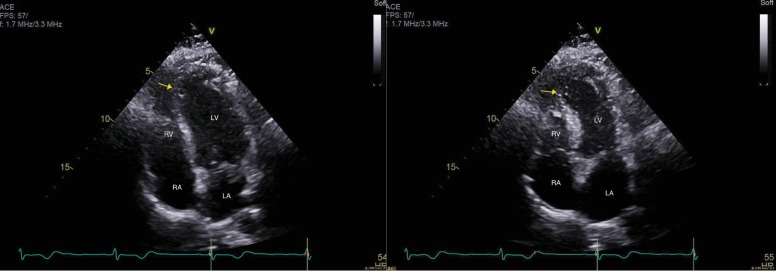
Transthoracic echocardiogram (TTE), revealing a hypokinetic cardiac apex. Yellow arrow on image of diastole (left) and systole (right) shows minimal apical wall motion during the cardiac cycle.

Further studies, including echocardiogram and nuclear stress testing, were pursued to better define her cardiovascular function. A transthoracic echocardiogram revealed an ejection fraction of 50%, suggestive of mild systolic dysfunction, in addition to the presence of apical hypokinesis (Figure 2). Grade II diastolic dysfunction was also noted. Her stress test confirmed an akinetic cardiac apex, as well as a small, severe-intensity, fixed defect, possibly suggestive of myocardial infarction. No areas of reversible ischemia were identified.

**Figure 3:**
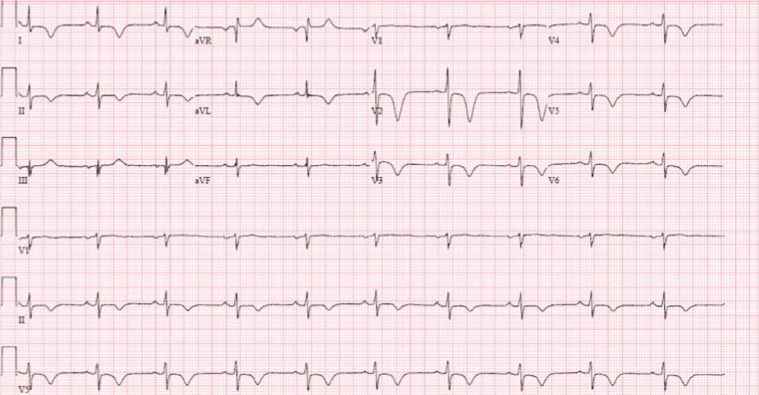
Repeat ECG again showing deeper T-wave inversions in leads V1-V3, with new T-wave inversions in leads I, II, aVL, and V3-V6.

After completing her stress test, the patient stated that her left-sided chest pain had returned and was accompanied by shortness of breath. A repeat ECG again showed deeper T-wave inversions in leads V1-V3, in addition to new T-wave inversions in leads I, II, aVL, and V4-V6 (Figure 3). A CT angiogram of her chest with IV contrast was able to rule out an underlying pulmonary embolism and aortic dissection as potential sources of her chest pain.

After further discussion with the patient and her family, she eventually agreed to undergo cardiac catheterization. The procedure established that all vessels were patent, effectively ruling out an acute ischemic event and atherosclerotic disease as the source of her symptoms. In the setting of left ventricular systolic dysfunction, the absence of coronary artery disease, and elevated cardiac enzymes with T-wave inversions on ECG, the patient was diagnosed with Takotsubo syndrome. Her postprocedure course was uncomplicated, and the patient was ultimately discharged home on aspirin 81 mg and simvastatin 20 mg for preventative purposes, with instructions to follow up for a repeat echocardiogram as an outpatient.

## 3. Discussion

Takotsubo syndrome is a condition often associated with immense emotional or physical stress. The mechanism through which this develops is still ill-defined but has been theorized to be associated with a surge of neuroendocrine hormones in response to the stress [1]. At least 1-2% of cases of acute coronary syndrome with elevated cardiac enzyme levels are linked to the condition [2], and patients are typically expected to provide a history of recent onset stressors, which may include events such as loss of family members or financial hardship, among others.

The classic clinical presentation of a patient with Takotsubo syndrome is often identical to true acute coronary syndrome, involving symptoms of acute-onset substernal chest pain, with associated symptoms of dyspnea and occasionally syncope. Diagnostic tools including ECG, cardiac biomarkers, echocardiogram, cardiac catheterization, and more recently the InterTAK Diagnostic Score also play a role in delineating potential sources of a patient's symptoms. Takotsubo syndrome may be associated with ECG findings of ST-segment elevations or depressions, QT-segment prolongations, and T-wave inversions. Cardiac biomarkers, such as serum troponin levels, are commonly elevated. Furthermore, echocardiogram will usually reveal left ventricular systolic dysfunction, most commonly with apical wall motion abnormalities in the form of akinesis, hypokinesis, or dyskinesis. This is typically paired with a hyperdynamic cardiac base. However, variant forms have also been identified, including midventricular and reverse subtypes. In the former, akinesis is noted in the midventricle and is seen with a hyperdynamic base and apex. In the reverse subtype, the cardiac base is akinetic, while the apex is hyperdynamic [3]. Cardiac catheterization can be useful in identifying the presence of obstructive coronary artery disease, but it is important to note that the presence of some atherosclerotic disease does not exclude the diagnosis. In the case of our patient, cardiac catheterization confirmed patent vasculature without atherosclerotic disease, suggesting the previously noted fixed defect on her stress test was attributable to attenuation artifact.

The management of Takotsubo syndrome is not yet well established and primarily focuses on supportive care [4]. The use of angiotensin-converting enzyme (ACE) inhibitors, angiotensin receptor blockers (ARBs), and beta-blockers has been suggested in cases involving heart failure; however, there are currently no guidelines or randomized studies supporting the use of these medications [4]. Regardless, all patients diagnosed with Takotsubo syndrome should have repeat echocardiograms to monitor their cardiac function and overall recovery [5]. This is particularly important, as the mortality rate during long-term follow-up has been reported to be 5.6% [6].

While our patient's presenting complaint of chest pain is expected in a patient with Takotsubo syndrome, the history she provided is not. She had undergone no significant emotional stressors in the recent past. Rather, she informed us that her symptoms began shortly after being injected with a sclerotherapy agent for the cosmetic treatment of varicose veins. The agent in question was polidocanol, marketed in the United States under the trade names Asclera® and Varithena®. The intravenous injection is formulated to irreversibly damage the endothelial lining of blood vessels, which in turn causes vessel occlusion through the formation of a network of platelets, fibrin, and cellular debris [7]. The occluded vessel is then ultimately replaced with connective tissue over time, leading to the desired effect of diminishing the prominence of vein varicosities. Although polidocanol typically works locally, systemic effects can be observed. To date, there has only been one documented case of Takotsubo syndrome developing after an injection of polidocanol [8], as well as one other involving a similar agent called sodium tetradecyl sulfate [9]. Our case report highlights the first documented case of likely polidocanol-induced Takotsubo syndrome occurring in the United States.

The overall purpose of this case report is to highlight a rare but clinically significant iatrogenic outcome of a chemical injection routinely used in the outpatient setting. While the complication of Takotsubo syndrome secondary to polidocanol use is uncommon, it is essential that those administering and receiving the intravenous injection be made aware that this diagnosis remains a clinically relevant adverse outcome that can mimic major ischemic cardiac events.
